# Room-temperature polariton quantum fluids in halide perovskites

**DOI:** 10.1038/s41467-022-34987-y

**Published:** 2022-11-30

**Authors:** Kai Peng, Renjie Tao, Louis Haeberlé, Quanwei Li, Dafei Jin, Graham R. Fleming, Stéphane Kéna-Cohen, Xiang Zhang, Wei Bao

**Affiliations:** 1 Department of Electrical and Computer Engineering, University of Nebraska-Lincoln, Lincoln, NE USA; 2 Nanoscale Science and Engineering Center, University of California, Berkeley, CA USA; 3 Department of Engineering Physics, École Polytechnique de Montréal, Montréal, QC Canada; 4 Department of Chemistry, University of California, Berkeley, CA USA; 5 Center for Nanoscale Materials, Argonne National Laboratory, Lemont, IL USA; 6 Faculty of Science and Faculty of Engineering, The University of Hong Kong, Hong Kong, China

**Keywords:** Quantum fluids and solids, Polaritons

## Abstract

Quantum fluids exhibit quantum mechanical effects at the macroscopic level, which contrast strongly with classical fluids. Gain-dissipative solid-state exciton-polaritons systems are promising emulation platforms for complex quantum fluid studies at elevated temperatures. Recently, halide perovskite polariton systems have emerged as materials with distinctive advantages over other room-temperature systems for future studies of topological physics, non-Abelian gauge fields, and spin-orbit interactions. However, the demonstration of nonlinear quantum hydrodynamics, such as superfluidity and Čerenkov flow, which is a consequence of the renormalized elementary excitation spectrum, remains elusive in halide perovskites. Here, using homogenous halide perovskites single crystals, we report, in both one- and two-dimensional cases, the complete set of quantum fluid phase transitions from normal classical fluids to scatterless polariton superfluids and supersonic fluids—all at room temperature, clear consequences of the Landau criterion. Specifically, the supersonic Čerenkov wave pattern was observed at room temperature. The experimental results are also in quantitative agreement with theoretical predictions from the dissipative Gross-Pitaevskii equation. Our results set the stage for exploring the rich non-equilibrium quantum fluid many-body physics at room temperature and also pave the way for important polaritonic device applications.

## Abstract (web summary)

Lead halide perovskites have recently emerged as a promising platform for the study of polariton superfluidity at room temperature. Here the authors report a complete set of quantum fluid phase transitions in both 1D and 2D homogeneous single crystals of CsPbBr_3_.

## Introduction

Quantum fluids, from superconducting electrons^1^ to superfluid helium^2^, from ultracold atoms Bose–Einstein condensation (BEC) on optical lattices^3^ to the cosmological-scale superfluid core in neutron stars^4^, embody exotic quantum behaviors emblematic of particles or excitations with bosonic statistics. The hydrodynamics of quantum fluids has been the source of tremendous interest in a multitude of many-body systems^1–3,5,6^. In particular, exciton-polaritons, quasiparticles composed of a superposition of the confined photons and excitons in a semiconductor microcavity, recently emerged as a unique driven-dissipative system for quantum fluid research^7^. These exciton-polaritons possess an ultra-small effective mass (~10^−5^ electron mass) from their photonic component and also inherit strong nonlinearity from their excitonic component. Thus, compared to ultracold atoms, polaritons can undergo BEC at elevated temperatures^8,9^, ultimately limited by the exciton binding energy. Indeed, a series of exciting observations of rich macroscopic quantum fluid phenomena were reported in exciton-polaritons condensation with the GaAs or CdTe quantum well microcavities at cryogenic temperatures, such as frictionless superfluidity and Čerenkov supersonic flow^10,11^, quantized vortices^12,13^, and soliton formation^14,15^. However, due to the small Wannier–Mott exciton binding energy of the III-V or II-VI materials, these experiments must still be performed at liquid helium temperatures. The low-temperature operation and expensive molecular-beam epitaxy materials growth significantly limit the accessibility and practical applications. Although high exciton binding energy materials such as GaN, ZnO, and two-dimensional semiconductors show great potential for room-temperature polaritonics^16–19^, they unfortunately still have limitations to overcome for studying the quantum fluid hydrodynamics.

Moreover, as a non-equilibrium system, some discussions were also involved in the research of polariton fluids^20–22^. Specifically, under continuous-wave (CW) coherent driving below the optical parametric oscillation (OPO) threshold^3^, recent theoretical work^21^ shows polariton fluids can only preserve some superfluid-like properties due to the phase locking and are better to be understood as a rigid state instead of superfluidity. In this aspect, pulsed-laser pumping work, where the phase of the system can subsequently evolve freely after excitations, has certain advantages^12,13,23^, in quantum fluids study and recently even shown polariton superfluidity at room temperature in non-crystalline organic microcavities^24^. However, the intrinsically small interactions of Frenkel excitons in organics, short polariton lifetimes, and structural inhomogeneity have hampered the observation of the Čerenkov wave pattern in the supersonic regime in accordance with the Landau criterion of superfluidity. Furthermore, due to the small polariton-polariton interactions in organics, the pumping power is also close to the material damage threshold^25,26^, making the system impossible to address higher density behavior at room temperature.

Recently, a new family of semiconducting materials, the lead halide perovskites with a composition of ABX_3_ (where A is commonly CH_3_NH_3_^+^ (MA^+^) or Cs^+^; B is Pb^2+^; X is Cl^−^, Br^−^, and I^−^), have attracted much attention because of their excellent optical performance in solar cells^27^ and optoelectronic devices^28,29^, thanks to the defect-tolerance, large carrier mobility, long lifetime, high photoluminescence (PL) efficiency, tunable bandgap, and simple single crystal growth processes^30–32^. Furthermore, single-crystalline bromide and chloride perovskites show excellent Wannier–Mott exciton properties for room-temperature polaritonics^33^, such as large exciton binding energy^33^, strong exciton photon coupling strength^34^, and high room-temperature nonlinear interaction strength^35^. With the chemical vapor deposition (CVD), small single crystal inorganic halide perovskites have demonstrated polariton condensation at room temperature^34^. Additionally, in contrast to conventional Wannier–Mott excitons materials like GaAs and CdTe, the intrinsic large and tunable splitting between transverse electric and transverse magnetic modes in perovskite cavities^36,37^ behaves as a winding in-plane magnetic field on the photon spin, enabling rich physics such as the Rashba–Dresselhaus Hamiltonians^38–41^, and spin textures^39,42,43^. However, the hallmark frictionless flow of nonlinear superfluidity and Landau criterion demonstration in halide perovskites, the foundation for studying rich spin-orbital coupled quantum fluid phenomena, remain elusive.

In this work, using large and extremely homogenous solution-grown halide perovskites^40^, we report the observation of polariton superfluidity in a Wannier-Mott exciton system at room temperature under resonantly pulsed excitations. Specifically, we demonstrate transitions from a normal fluid to superfluidity and supersonic fluid in both one- and two-dimensional cases in halide perovskite microcavities. In the one-dimensional case, we show that back-reflection can be fully suppressed outside the superfluidity healing length when the polariton superfluid hits a hard potential wall under the critical velocity. In the two-dimensional case, we demonstrate landmark zero-viscosity superfluid and a Čerenkov supersonic wave pattern at room temperature, on par with low-temperature cases^11^. The experimental data are also in quantitative agreement with our theoretical modeling using non-equilibrium Gross-Pitaevskii equations. Lastly, by comparing the simulations and experimental real-space images, the polariton-polariton nonlinear interaction strength from the inorganic perovskite’s Wannier-Mott exciton was also extracted to be more than two orders of magnitude higher than the Frenkel exciton in organics^24^. Our work reveals that halide perovskites microcavities can enable previously inaccessible complex quantum fluid behaviors at room temperature. It also creates a transformative room-temperature polariton playground with Rashba-like dispersion^39^ for exciting topological quantum fluid studies.

## Results

**Fig. 1:**
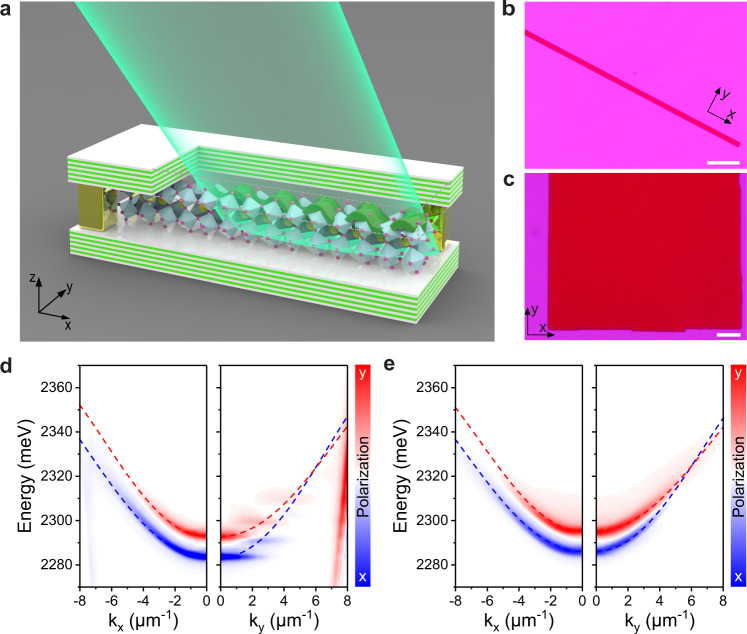
Sketch of experimental configuration and characterizations of CsPbBr_3_ microwire and plate. **a** Schematics of polariton fluid experiments. The polariton fluid was resonantly pumped by a fs pulse laser, and the signal was detected in the transmission configuration (Supplementary Fig. 4). **b**, **c** are the optical images of a perovskite microwire and a plate under transmission configuration, respectively. Scale bar: 20 μm. **d**, **e** are the linear-polarized dispersions of photoluminescence (PL) of a perovskite microwire with a width of 1.85 μm and a plate along the two perpendicular crystal axes (*x*- and *y*-axis shown in Fig. b and c). Due to the confinement, the discrete energy levels along the *y*-axis of the microwire can be observed. Meanwhile, the spin-orbital coupling effect induces the two dispersion branches to cross along the *y*-axis. The dashed lines are the fittings of the two lower branches. The details of the fitting are shown in Supplementary Note 1. The two lower polariton branches with polarization along the *x*- (blue) and *y*- (red) axis cross at the diabolic points at *k*_*y*_ = 5.9 μm^−1^.

Our microcavity samples^40^ were made by directly solution-synthesizing the CsPbBr_3_ (exciton binding energy ~36 meV^44^) single crystals inside the prefabricated empty optical cavities, which were formed by a unique wafer-bonding process. Chemical synthesis under nanometer-size confinement enables highly homogenous single-crystalline perovskite microwires and plates with substantial sizes (Fig. 1), without any sample degradation due to the top DBR fabrication.

Firstly, a one-dimensional perovskite microwire was used to study superfluidity. The recent one-dimensional experiment in GaAs emulated the black hole and Hawking radiation with polariton superfluidity at low temperature^45^. However, despite its importance in polariton experiments^17,46,47^, there are no previous demonstrations of the one-dimensional superfluidity at room temperature. Figure 1b shows a long CsPbBr_3_ single-crystal microwire. Figure 1d shows the angle-resolved PL of a perovskite microwire with a width of 1.85 μm. Along the long *x*-axis, the angle-resolved photoluminescence shows the two continuous lower polariton branches (The details of the fitting are shown in Supplementary Note 1), which are split at *k*_||_ = 0 due to the optical birefringence from the orthorhombic phase of CsPbBr_3_. In contrast, the lateral confinement along the short y-axis induces discrete energy levels in the dispersion (Fig. 1d). More interestingly, the mode splitting can lead to tunable Rashba-like dispersions^39–41^, highly desired for synthetic non-Abelian gauge fields and topological physics studies.

**Fig. 2:**
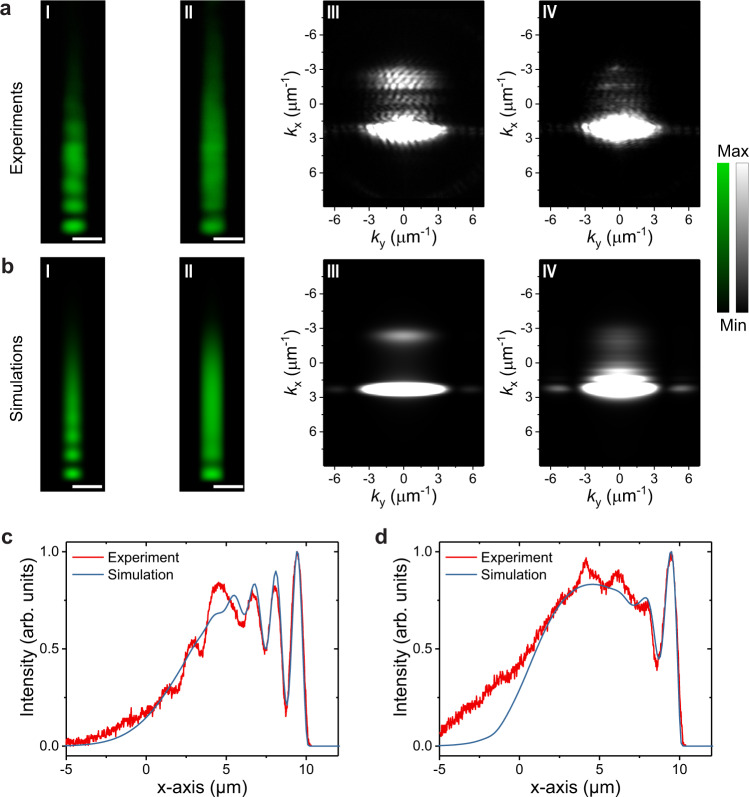
Room-temperature demonstration of one-dimensional polariton superfluid transition. **a**-I, **a**-II Experimental real-space images of polariton fluid with resonance pumping power in the cavity of 0.04 (I) and 3.8 (II) μJ cm^−2^, respectively. The polariton fluid with injection momentum of *k*_||_ = 2.3 μm^−1^ flows along the microwire and is reflected by the edge, showing interference patterns in real-space (**a**-I) at low polariton density. While at high polariton density, the polariton fluid entered the superfluid regime, and the interference pattern vanished outside the healing length region. **a**-III, **a**-IV The experimental saturated images of momentum distributions corresponding to **a**-I and **a**-II, respectively. The back reflection signals were clearly observed at low polariton density and suppressed at high polariton density. The residual scattering signals at the superfluidity condition are due to the polariton mode broadening induced non-resonant excitation^49^ and time-averaged imaging under pulse excitation. **b**-I, **b**-II The time-averaged simulations in real-space before and after the transition. **b**-III, **b**-IV The simulated saturated time-averaged images in momentum space corresponding to **b**-I and **b**-II, respectively. **c** (**d**) The extracted normalized experimental and simulated real-space profiles along the long axis from **a**-I (**a**-II) and **b**-I (**b**-II) with low (high) polariton densities. The simulations fit the experimental results very well. The inconsistency between 1-6 μm is due to the small sample inhomogeneity. All scale bars: 2 μm.

Here, a linear-polarized pulsed laser was used to only excite one polariton branch resonantly to inject polariton fluid flowing along the long x-axis toward the edge of the microwire in a transmission configuration (Fig. 1 and Supplementary Fig. 4). The resonant excitation can easily generate propagating polariton fluid with designed in-plane momentum *k*_||_ compared with the non-resonant excitation and the triggered optical parametric oscillator^10,23^. In Landau’s theory of superfluidity, spontaneous energy dissipation or scattering can occur in quantum fluids only if it can reduce the energy of the moving fluid. Robust superfluidity with zero viscosity can exist if the quantum fluid’s group velocity *v*_p_ is smaller than the critical speed *v*_c_ of the system (Landau criterion *v*_p_ < *v*_c_). As for a polariton fluid, the critical velocity *v*_c_ is the polariton speed of sound ${c}_{s}=\sqrt{g|{\psi }_{p}{|}^{2}/m}$^11,48^. Experimentally, the injected polariton fluid group velocity ${v}_{p}=\hslash {k}_{p}/m$ can be tuned by adjusting the in-plane wavevector *k*_p_, i.e., changing the incident angle (Supplementary Fig. 4). In these two expressions, *g* describes the polariton-polariton interaction, $|{\psi }_{p}{|}^{2}$ is the polariton density, *m* is the polariton effective mass, and $\hslash$ is the reduced Planck constant. A small *k*_p_ = 2.3 µm^−1^ was first chosen to satisfy the subsonic requirement of *c*_s_ > *v*_p_. By tuning the polariton densities with the pumping power, the speed of sound can be tuned to fulfill the subsonic condition of superfluidity. At low excitation power, where the polariton density is low, the polariton fluid was clearly reflected back by the edge (a “hard” potential wall for the polariton fluid). As a result, the interference fringes between the incoming fluid and reflected fluid can be observed (Fig. 2a-I), accompanied by bright back reflection signals in momentum space at *k*_x_ = 2.3 µm^−1^ (Fig. 2a-III). At high excitation power, the system enters the superfluidity regime (due to the increased speed of sound), and interestingly, the interference fringes in real space and the back reflection signals in momentum space disappear (Fig. 2a-II, a-IV). Since the polariton superfluid cannot go through the microwire’s end, the back reflection from the end will inevitably occur. In a microscopic picture, this back-reflection will eventually be suppressed through the polariton-polariton nonlinear interaction (Supplementary Fig. 6) outside the superfluidity healing length region (Supplementary Fig. 7 for CW excitation case). All the excitations only occur inside the healing length region where the bulk superfluidity condition breaks down. Thus, we can extract from the experimental data (Fig.  2c, d) that the healing length is ~3 µm in the fs laser pump case (similar for CW case as shown in Supplementary Fig. 7). These results and physical picture are also in quantitative agreement with our Gross-Pitaevskii equations modeling (Supplementary Note 2, Supplementary Fig. 7) both in real-space (Fig. 2b-I, b-II, c, d) and momentum space (Fig. 2b-III, b-IV). Lastly, the small non-uniform intensity profile and residual interference fringes at the superfluidity condition outside the healing length region are also due to the Gaussian shape fs-pulsed excitation, time-average imaging, the mode broadening induced by non-resonant excitation^49^, as well as small sample inhomogeneities.

**Fig. 3:**
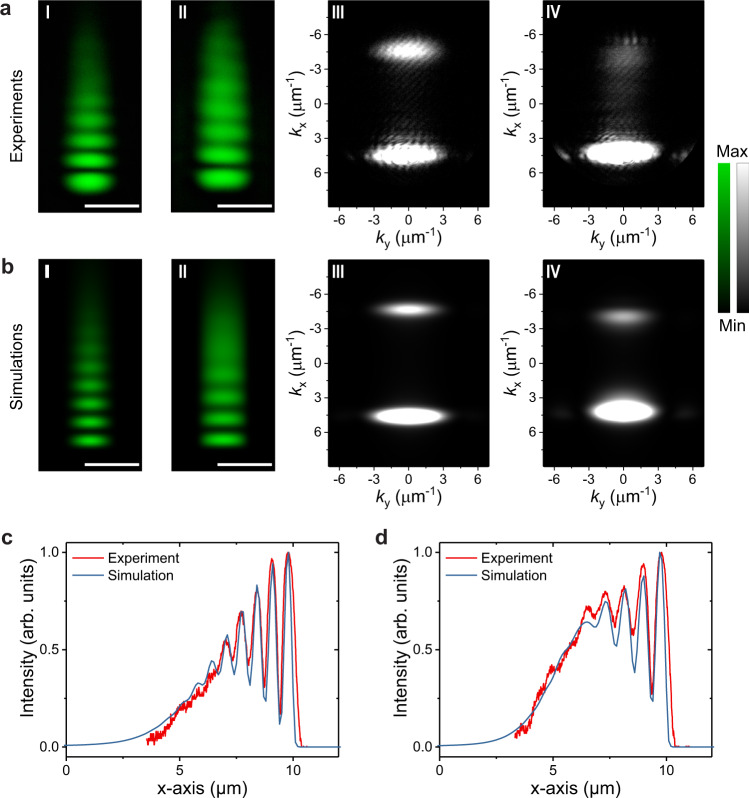
Room-temperature demonstration of one-dimensional polariton supersonic regime. **a**-I, **a**-II (**a**-III, **a**-IV) Experimental real-space (momentum-space) images of polariton fluid with resonance pumping power in the cavity of 0.08 (I) and 6.76 (II) μJ cm^−2^, respectively. With larger injection momentum of 4.6 μm^−1^, the polariton fluid was still in supersonic regime at high polariton density, as shown by the interference fringes and scattering signals in **a**-II and **a**-IV. **b**-I, **b**-II (**b**-III, **b**-IV) Numerically simulated real-space (momentum-space) images of polariton fluid corresponding to **a**-I, **a**-II (**a**-III, **a**-IV). **c** (**d**) The extracted normalized experimental and simulated real-space profiles along the long axis from **a**-I (**a**-II) and **b**-I (**b**-II) with low (high) polariton densities. The simulations fit the experimental results very well. The experiment was performed on the same microwire shown in Fig. 2. All scale bars: 2 μm.

When the polariton fluid is injected at a high *k*_p_ (4.6 µm^−1^) to fulfill supersonic condition *c*_s_ < *v*_p_, superfluidity will be destroyed based on the Landau criterion. Even at a very high polariton density, the interference fringes in real space and back-reflection signals in momentum space can always be ambiguously observed (Fig. 3a). Meanwhile, due to the increased nonlinear interactions between polaritons at high density, the wavenumber of the polariton fluid flow *k* decreases slightly to 4.2 µm^−1^ at high pumping intensity, as indicated by the increasing interference fringe distances (Fig. 3c, d). A similar decreased *k* effect was also reported in the previous black hole and Hawking radiation experiments at low temperature^45^. The Gross-Pitaevskii equations simulations again confirm these observations nicely, as shown in Fig. 3b–d.

**Fig. 4:**
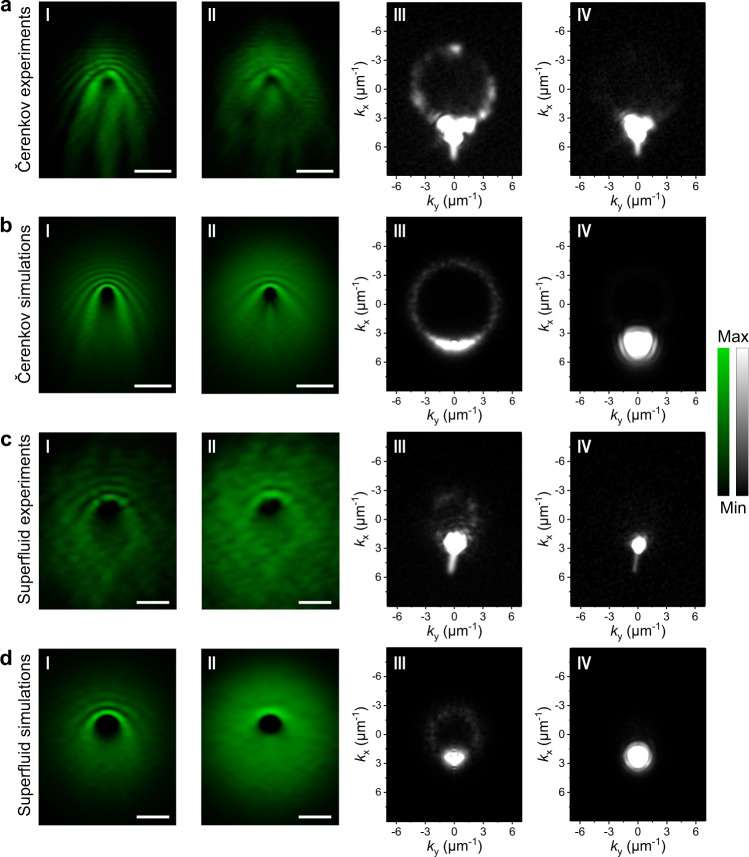
Demonstration of two-dimensional full polariton hydrodynamic transitions from a normal fluid to both a Čerenkov supersonic and a superfluid fluid. **a**-I, **a**-II Experimental real-space images of polariton flowing with injection momentum of *k*_||_ = 4.2 μm^−1^ across a defect with resonance pumping power in the cavity of 0.07 (I) and 6 (II) μJ cm^−2^, respectively. **a**-III-IV, The experimental saturated images of momentum distributions corresponding to **a**-I and **a**-II, respectively. At low polariton density (**a**-I, **a**-III), the scattering by the defect generated parabolic interference wavefronts and Rayleigh scattering ring. When polariton density increased, Čerenkov behavior in the supersonic regime (c_s_ < v_p_) was shown as the linear wavefronts and the increased aperture angle. The scattering ring gets modified at high polariton density (**a**-IV). **b**-I, **b**-II, the time-averaged simulation in real-space before and after the transition, respectively. Evenly distributed 0-5 meV exciton energy random variations are added to only 0.3% of the spatial pixels to better fit the experimental results. **b**-III, **b**-IV The time-averaged simulation images of the momentum space corresponding to b-I and b-II, respectively. **c**-I, **c**-II (**c**-III, **c**-IV) Experimental real-space (momentum space) images of polariton flowing with injection momentum of *k*_||_ = 2.3 μm^−1^ with resonance pumping power in the cavity of 0.06 (I) and 5.6 (II) μJ cm^−2^, respectively. At high polariton density (**c**-II, **c**-IV), the polariton fluid entered the frictionless superfluid regime and the interference fringes and scattering ring vanished. **d**-I, **d**-II (**d**-III, **d**-IV) The simulated time-averaged real-space (momentum space) simulations before and after the transition. Evenly distributed 0-5 meV exciton energy random variations are added to only 1% of the spatial pixels to better fit the experimental results. Compared to simulations, the additional short white lines at the bottom of the **a**-III, **a**-IV and **c**-III, **c**-IV images are streak artifacts caused by the scanning of the CCD camera. More power-dependent experimental images of Čerenkov and superfluid cases were shown in Supplementary Figs. 8–12. All scale bars: 5 μm.

Another evidence of the polariton fluid supersonic regime is the increasing Mach cone angle with Čerenkov wave pattern when polariton fluid flows across a defect in a two-dimensional configuration (Fig. 1c). However, in previous work with organics^24^, the linear Čerenkov wave pattern was not demonstrated when the polariton fluid was excited at a large *k*_p_. Moreover, a clear change of the scattering cone with increasing density was not observed due to the limited range of powers achievable in that system (Supplementary Fig. 15). Here, with the perovskite microcavities, parabolic wavefronts due to the interference around the defect were clearly observed at low polariton density (*k*_p_ = 4.2 μm^−1^, Fig. 4a-I), accompanied by a bright Rayleigh scattering ring in momentum space (Fig. 4a-III). With increased pumping power, the parabolic wavefronts across the defect became linear, and the aperture angle gradually increased (Fig. 4a-II and Supplementary Fig. 8). The Mach cone is formed due to the polariton density modulation downstream (Fig. 4a-II), which was also well reproduced in the simulation nearly perfectly (Fig. 4b-I, a-II). Meanwhile, the scattering ring in the momentum space was heavily modified, as shown in Fig. 4a-III, a-IV. This observation of the Čerenkov pattern at room temperature can directly prove the existence of a well-defined sound velocity in the polariton fluid with perovskite in contrast to previous organics cases. The averaged sound velocity was also extracted as *c*_s_ = 10.4 μm ps^−1^ from Fig. 4a-II by $\sin (\phi )={c}_{s}/{v}_{p}$, where $\phi$ is the half Mach cone angle^11,50^. Lastly, the two dark streaks in the wake of a defect (Fig. 4a-II, b-II) are most likely caused by oblique dark solitons^7,14^, an effect that has never been observed at room temperature and is worthy of further investigation.

Finally, the superfluidity in two-dimensional perovskite microcavity was also observed with a smaller injection momentum at *k*_p_ = 2.3 μm^−1^ (Fig. 4c). At high polariton density (Figs. 4c-II, c-IV and 5b), the interference parabolic wavefront and the Rayleigh scattering ring were strongly suppressed, illustrating a “frictionless” superfluidity behavior. The simulations in Fig. 4d match the experiment very well.

**Fig. 5:**
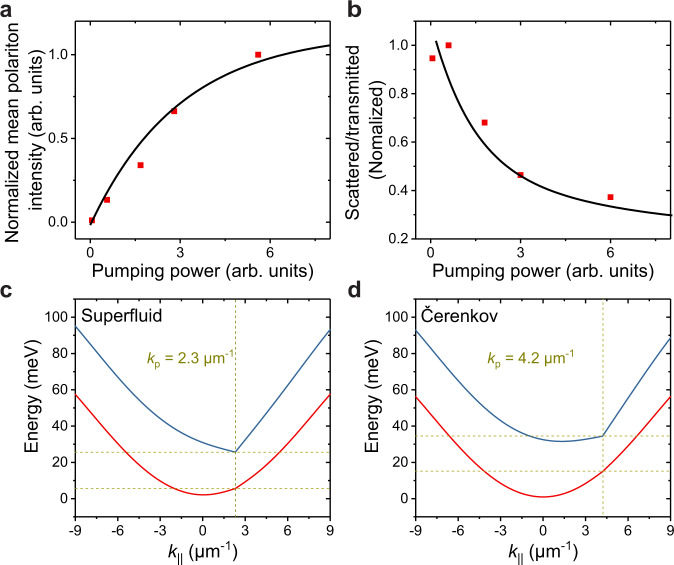
Power-dependence of the superfluidity and simulated Bogoliubov excitation spectra of the lower polariton branch. **a** Mean polariton density (normalized) as a function of pumping power in the superfluid regime. **b** Ratio of scattered light to transmitted light (normalized) as a function of pumping power in the superfluidity regime. The experimental data (red solid points) are obtained from the scattering ring in Fig. 4c-III, c-IV and Supplementary Fig. 9. The black solid lines are the simulated results in both **a** and **b**. **c** Positive Bogoliubov excitation spectra correspond to the superfluidity case in Fig. and 4c, d with *k*_p_ = 2.3 μm^−1^. The red and blue solid lines correspond to the low and high pumping power cases, respectively. Bogoliubov excitation spectra are a good model even for the fs-laser pumping cases, as previously demonstrated^12,24^. Here the superfluidity regime was analyzed. At low pumping power, the dispersion shows a parabolic shape, and the injected polariton can be elastically scattered to the same energy states as indicated by the yellow dashed lines. At high pumping power, the polariton-polariton interaction induces a strong blueshift and tilts the dispersion at high *k*, and a discontinuity emerges in the excitation spectrum. As a result, there will be no states available to be scattered at the energy of the injected polariton. Thus, the system enters the superfluidity regime. The corresponding averaged polariton densities is 3.3 × 10^4^ pol μm^−2^ obtained from Fig. 4d-II for superfluidity case. **d** Positive Bogoliubov excitation spectra correspond to the Čerenkov case in Fig. 4a, b with *k*_p_ = 4.2 μm^−1^. At the high polariton density case, though strong blueshift reappears, there are still states with energy at and below the pumping energy as indicated by the yellow dashed lines. As a result, the system is in the supersonic case and shows a Čerenkov wave pattern. The sound velocity can be extracted as 10.1 μm ps^−1^ here, consistent with the value of 10.4 μm ps^−1^ extracted from the experimental Mach cone angle in Fig. 4a-II. The corresponding averaged polariton densities is 3.4 × 10^4^ pol μm^−2^ obtained from Fig. 4b-II.

The complex polariton fluid behaviors in superfluid and Čerenkov regimes can also be qualitatively understood by the Bogoliubov excitation spectra analysis^49^ (Fig. 5c, d). It is also worth noting that pulsed excitation rather than the CW excitation was chosen in this experiment. Since there is no stationary state under pulsed pumping, the observation of polariton fluid evolution is a time-averaged effect of different Bogoliubov states. Nevertheless, the main feature of the superfluidity and the Čerenkov state can be qualitatively captured by the Bogoliubov excitation spectra. For the superfluidity case in Fig. 5c, at low pumping power, the dispersion shows a parabolic shape (the solid red line), and the injected polariton can be elastically scattered to the same energy states as indicated by the yellow dashed lines. At high pumping power with high polariton density, the polariton-polariton interaction induces a strong blueshift and tilts the dispersion at high k, and a discontinuity emerges in the excitation spectrum (the solid blue line). As a result, there will be no states available to be scattered at the energy of the injected polariton. Thus, the elastic scattering vanishes, and the system enters the superfluidity regime. While for the Čerenkov case in Fig. 5d, at high pumping power, though strong blueshift reappears, there are still states with energy at and below the pumping energy as indicated by the yellow dashed lines. As a result, the system is in the supersonic case and shows a Čerenkov wave pattern.

## Discussion

It is worth noting that, the phase-locking effect in the CW resonant excitation^21^ does not exist in pulsed excitation cases because the polariton can freely evolve for a significant proportion of its life after the laser pulse excitation (Supplementary Fig. 13). Furthermore, the generation and flow of vortex pairs can be observed behind the defect in the supersonic regime in previous experimental reports^12,13,24^, a signature of phase evolution after the laser pulse. On the other hand, pulsed excitation also helps to reduce harmful thermal effects on the perovskite samples. Therefore, the observations were a time-averaged effect of the evolutions of different states (Supplementary Figs. 7 and 13).

We emphasize that strongly interacting polaritons are crucial for defining the sound velocity and stabilizing the polariton condensation and superfluidity (Supplementary Fig. 6). The interaction constant *g* in these experiments can be extracted (Supplementary Note 2) as *g*_xx_ ~ 4 μeV μm^2^ (between excitons) and *g*_pp_ ~ 0.5 μeV μm^2^, (between polaritons) from real-space images fittings as well as the sound velocity formula in the Čerenkov experiment *c*_*s*_. The polariton-polariton interaction strength is thus more than two orders of magnitude higher than organics^24^, and is also comparable with those in the low-temperature GaAs^51^ and other perovskite works at room temperature^35,52^. This suggests an intrinsic strong polariton-polariton interaction and high nonlinearity attributable to the Wannier-Mott exciton. In contrast, weakly interacting Frenkel excitons in organics demand a much stronger excitation laser pulse, which is close to the organic material’s photo-damaging threshold or has to go beyond it^24–26^. Lastly, our room-temperature two-dimensional experimental image quality is comparable to the low-temperature MBE-grown GaAs case^11^, which further justifies the excellence of the halide perovskite platform.

To conclude, we report the room-temperature polaritonic quantum fluid behaviors in solution-synthesized halide perovskites microcavities in both one- and two-dimensional cases. Room-temperature polaritonic quantum fluid phase transitions from a normal fluid to both superfluid and (Čerenkov, if two-dimensional) supersonic fluid were demonstrated. Our work established a robust room-temperature quantum fluid platform with strongly interacting polaritons and paved the way for a transformative playground for studies, such as non-equilibrium topological physics^53,54^, non-Abelian gauge fields^39^, and non-equilibrium collective excitation spectrum^55–57^. The quantum fluid study presented here can also guide future quantum fluid theory work on pulsed laser excitation cases and the development of other room-temperature polariton experiments such as layered perovskite materials^35^, and other types of cavities like DBR-free open cavity configurations^58,59^. The strong nonlinearity originating from halide perovskites’ Wannier–Mott excitons could also lead to other device applications, such as topological polaritonic laser and non-Hermitian photonic devices^60^.

## Methods

### Fabrication of optical cavity and nanocavity

#### Fabrication of distributed Bragg reflector (DBR) wafer

Quartz wafers were cleaned by heated piranha baths followed by deionized water washes. The cleaned wafers were loaded into a vacuum chamber for SiO_2_/Ta_2_O_5_ deposition by electron beam evaporation with an advanced plasma source. As a result, nine pairs of the SiO_2_/Ta_2_O_5_ DBR mirror were deposited at 300 °C. The elevated temperature and high-kinetic energy plasma bombardments produced an enhanced optical quality of the DBR mirrors.

#### Thermal compression wafer-wafer bonding of DBR wafer with patterned gold pillar

A two-dimensional array of squared gold pads was deposited on the surface of DBR wafers by electron beam evaporation. Then two gold-patterned wafers were loaded into the wafer bonder. The wafers were aligned according to the gold pads, followed by a thermal compression bonding process. Finally, the bonded wafer was diced into small chips for crystal growth. Bonded DBR chips are shown in Supplementary Fig. 1.

### Synthesis of halide perovskites

#### Growth of all CsPbBr_3_ crystals in the nanocavity

We used a inverse temperature crystallization (ITC). Dimethyl sulfoxide (DMSO) was added into cesium bromide and lead bromide powder mixture in a vial. All chemicals were purchased from Sigma-Aldrich Chemical and used as received. The prepared solution was dropped at the edge of the fabricated DBR nanocavity, and the cavity space was fully filled by the solution through capillary force. The DBR nanocavity with CsPbBr_3_ precursor solution was put on a hotplate for crystal growth. After that, the DBR nanocavity with crystals was put in a vacuum chamber to remove possible residual solvent.

### Characterizations of halide perovskites

#### Atomic force microscopy (AFM) measurements

Nanocavities were opened by brute force with tweezers to expose perovskite crystals before AFM measurements. The AFM images and height profiles of crystals were taken with Park Systems AFM in tapping mode and analyzed with the XEI software.

#### PL lifetime measurements

The PL lifetime was measured using a home-built multifunctional microscope integrated with a time-correlated single-photon counting system. Femtosecond pulsed laser centered at 808 nm (Coherent Mira 900-F) was used for second harmonic generation at 404 nm via a barium borate crystal, and the produced 404 nm laser was fiber-coupled to the microscope and focused on the samples through an objective lens. The PL was collected by the same objective and fiber-coupled to a single-photon counting module (Excelitas SPCM AQRH 13) after blocking the 404 nm excitation. The PL photon counts were then time-correlated (IDQuantique ID 900) with the sync signal from the pulsed laser to build the histogram of photon arrival time, from which PL lifetime can be extracted. Nanocavities were opened before lifetime measurements.

### Polariton fluid measurements

The polariton fluid measurements were performed using a home-built transmission set-up at room temperature. A linear-polarized 250-fs optical parametric amplifier pulse laser with a repetition rate of 20 kHz was used to achieve resonant excitation of one lower polariton branch. Due to the broad linewidth (~3.5 nm (14.8 meV) at FWHM) of the pulse laser, the resonant can be easily achieved. The center energy of the laser (~2.3 eV) was also a little blue shifted to achieve resonant excitation of the renormalized dispersions caused by polariton-polariton interactions at high polariton density. The laser pulse was focused on the sample by a ×20 microscope objective (Nikon Plan Fluor ELWD) with NA = 0.45. The laser beam was shifted to tune the incident angle. The diameter of the focused laser beam was around 15 μm. From the other side of the microcavity, a ×40 (NA = 0.6) or a ×60 (NA = 0.7) objective (Nikon Plan Fluor ELWD) was used to collect the signal. The images in real and momentum space were observed simultaneously by a CCD camera and an Andor spectrometer equipped with a 2D EMCCD camera, respectively. A Fourier imaging configuration was used to obtain *k*-space imaging. The experimental set-up for superfluidity measurement is shown in Supplementary Fig. 4. A polarizer and a half waveplate were used in the excitation to pump only one branch and used in the collection to obtain the polarization-resolved PL dispersions.

The defects in 2D hydrodynamic measurements were burned artificially by non-resonantly fs-pumping the sample beyond the damage threshold power density (>20 times the polariton condensation transition threshold). The repetition rate of the 250-fs optical parametric amplifier pulse laser was set to 200 kHz to enhance the heat effect during this process. The laser was focused on the sample with a ×40 (NA = 0.6) or a ×60 (NA = 0.7) Nikon objective. The diameters of the defects were about *d* = 1-4 μm.

## Supplementary information

Supplementary Information

## Data Availability

The authors declare that the main data supporting the findings of this study are available within the paper. Extra data are available from the corresponding authors upon reasonable request. Source data are provided with this paper.

## Source data

Source Data
