# Supplementary material for: Room-temperature polariton quantum fluids in halide perovskites
Source: Nat Commun. 2022 Nov 30;13:7388. doi: 10.1038/s41467-022-34987-y (PMC9712507; doi:10.1038/s41467-022-34987-y)
Supplement: Supplementary file 1 — Supplementary Information [file 41467_2022_34987_MOESM1_ESM.pdf]

Supplementary Information for

Room-temperature polariton quantum fluids in halide perovskites

Kai Peng[1]*, Renjie Tao[2]*, Louis Haeberlé[3], Quanwei Li[4], Dafei Jin[5], Graham R. Fleming[4], Stéphane Kéna-Cohen[3], Xiang Zhang[2,6†], and Wei Bao[1†]

*These authors contributed equally to this work.
†Corresponding author. Email: wbao@unl.edu or xiang@berkeley.edu

[1]Department of Electrical and Computer Engineering, University of Nebraska-Lincoln, Lincoln, Nebraska, United States

[2]Nanoscale Science and Engineering Center, University of California, Berkeley, California, United States

[3]Department of Engineering Physics, École Polytechnique de Montréal, Montréal, Quebec, Canada

[4]Department of Chemistry, University of California, Berkeley, California, United States

[5]Center for Nanoscale Materials, Argonne National Laboratory, Lemont, Illinois, United States

[6]Faculty of Science and Faculty of Engineering, The University of Hong Kong, Hong Kong, China

This PDF file includes:

Supplementary Text
Supplementary Figs. 1 to 15

Supplementary Text

Supplementary Note 1: Polariton model

The polariton dispersion was fitted using a coupled oscillator model neglecting linewidths:

$$\begin{pmatrix} E_{cav}(k) & \Omega/2 \\ \Omega/2 & E_{ex} \end{pmatrix}\begin{pmatrix} a \\ b \end{pmatrix} = E\begin{pmatrix} a \\ b \end{pmatrix}.$$

Here, the cavity mode is represented as $E_{cav}(k) = E_0 + \frac{\hbar^2 k^2}{2m}$ with cutoff photon energy E_0 and effective mass m. E_{ex} is the exciton energy of the sample. E is the eigenvalue of the polaritons, and a and b represent the Hopfield coefficients satisfying $|a|^2 + |b|^2 = 1$.

Meanwhile, the spin-orbit coupling induces TE-TM mode splitting of the planar microcavity, which has two polarized modes with different effect masses m_{TE} and m_{TM}. The material birefringence in the cavity breaks the cylindrical symmetry of TE and TM modes and lifts their degeneracy at k = 0. The difference of the refractive index along the two crystal axes is estimated as about 1%. This spin-orbit coupling can be described on a circular polarization basis with parabolic approximation as [2,3]:

$$H_k = \begin{pmatrix} E_0 + \frac{\hbar^2 k^2}{2m} & \alpha - \beta k^2 e^{-2i\varphi} \\ \alpha - \beta k^2 e^{2i\varphi} & E_0 + \frac{\hbar^2 k^2}{2m} \end{pmatrix}$$

where $m = m_{TE} m_{TM}/(m_{TE} + m_{TM})$ is the reduced effective mass; $k = |k_{\parallel}| = \sqrt{k_x^2 + k_y^2}$ is the in-plane wavevector ($k_x = k\cos\varphi$, $k_y = k\sin\varphi$, φ is the propagation angle). α and β represent the optical birefringence from anisotropy and TE-TM splitting from spin-orbital coupling, respectively. Along y-direction ($\varphi = 0$), the two polariton branches cross at $k_y = \pm\sqrt{\frac{\alpha}{\beta}}$ as the two diabolical points. With consideration of the strong coupling with the exciton, the Hamiltonian can be written as:

$$H_k = \begin{pmatrix} E_0 + \frac{\hbar^2 k^2}{2m} & \frac{\Omega}{2} & \alpha - \beta k^2 e^{-2i\varphi} & 0 \\ \frac{\Omega}{2} & E_{ex} & 0 & 0 \\ \alpha - \beta k^2 e^{2i\varphi} & 0 & E_0 + \frac{\hbar^2 k^2}{2m} & \frac{\Omega}{2} \\ 0 & 0 & \frac{\Omega}{2} & E_{ex} \end{pmatrix}$$

The two lower polariton branches in Fig 1c along the x and y-axis were fitted well with this model. Here, exciton energy extracted from the absorption spectrum (Fig. S2a) as E_{ex} = 2406 meV. The parameters in Fig. 1d were fitted as E_0 = 2340.44±12.2 meV, m = 0.14±0.01 meV/(μm/(ps))^2, α = 6.8±0.6 meV, β = 0.19±0.02 meV μm^2, Ω = 156.89±18.06 meV. The parameters in Fig. 1e were

fitted as E_0 = 2342.54±16.6 meV, m = 0.14±0.02 meV/(μm/(ps))^2, α = 6.62±0.8 meV, β = 0.18±0.02 meV μm^2, Ω = 154.54±24.44 meV.

Supplementary Note 2: Gross-Pitaevskii equations simulation

In the polariton fluid experiments, only one branch was pumped resonantly. So, we can just consider a simple coupled oscillator model by ignoring the spin-orbital coupling in the simulations. The nonequilibrium Gross-Pitaevskii equations were solving numerically [4,5]:

$$i\hbar \frac{d}{dt}\begin{pmatrix}\psi_C(\mathbf{x},t)\\ \psi_X(\mathbf{x},t)\end{pmatrix} = \begin{pmatrix}F(\mathbf{x},t)\\ 0\end{pmatrix} + \left[h^0 + \begin{pmatrix}-i\hbar\frac{\gamma_C(\mathbf{x})}{2} & 0\\ 0 & V_X(\mathbf{x}) - i\hbar\frac{\gamma_X}{2} + g_X\left|\psi_X(\mathbf{x},t)\right|^2\end{pmatrix}\right]\begin{pmatrix}\psi_C(\mathbf{x},t)\\ \psi_X(\mathbf{x},t)\end{pmatrix}$$

where the single-particle Hamiltonian is given by

$$h_0 = \hbar\begin{pmatrix}\omega_C(-i\nabla) & \Omega_R\\ \Omega_R & \omega_X(-i\nabla)\end{pmatrix}.$$

The polariton dispersion parameters were chosen as E_{ph} = 2334.9 meV and E_{ex} = 2406 meV, which responds to a detuning of Δ = -71.1 meV. The effective mass was set as 0.16 meV/(μm/(ps))^2 by fitting the TE polariton lower branch with coupled oscillator model neglecting spin-orbital coupling and the Rabi splitting was set as Ω_R = 151.4 meV. $\psi_{C(X)}$ is the photon (exciton) wavefunction. The photon decay rate γ_C is set as 3.9 ps^{-1}, which was estimated by the Q of the cavity (~900). The exciton decay rate γ_X is set as 1.25×10^{-4} ps^{-1}, which was estimated by the lifetime measurement in Fig. S2b.

The region outside the microwire and the defects in two-dimensional simulations were introduced by setting the exciton potential $V_X(\mathbf{x})$ and the photon decay distribution $\gamma_C(\mathbf{x})$. The burning defect potential and region outside the microwire for the exciton was set to be 50 meV, and the photon decay rate was set 200 times larger. A uniform random distribution between 0-5 meV exciton potential fluctuation was introduced to account for the PL inhomogeneous shift. The lateral length scale of the disorder is set as the simulation grid step of 0.1 μm (each pixel size).

The polariton was pumped by a 250-fs pulsed laser with a Gaussian spot:

$$F(\mathbf{x},t) = \hbar F_p e^{i(\mathbf{k}_\mathrm{p}\cdot\mathbf{x}-\omega_p t)} e^{-\frac{\mathbf{x}^2}{2w_x^2}} e^{-\frac{(t-t_0)^2}{2\sigma_t^2}}.$$

The laser was set to pump the cavity photon directly, and its amplitude can be related to the incident pump intensity according to input-output theory[6] as:

$$F_p = \sqrt{\frac{\gamma_C I_0}{2\hbar\omega_p}}.$$

The pumping fluence in the cavity was obtained using $E_{in} = I_0\sigma_t\sqrt{\pi}$. The effective pumping fluences inside the cavity I_0 in the simulations are set as 26 μJ cm^{-2} in the superfluid and supersonic regime in Fig. 2-5, consist with the experimental value of ~3.8-6.78 μJ cm^{-2}. The pumping laser energy is set ~5 meV blueshift than the lower polariton branch at the injection momentum. The

pumping power density in the experiments is an approximate averaged value of a gaussian laser spot. Therefore, the pumping power densities in the simulation were chosen with a discrepancy to fit the experiments.

The polariton-polariton interaction was introduced by setting the exciton-exciton interaction term. The exciton-exciton interaction constant g_X is chosen as 4 μeV μm^{-2} in the simulation, which corresponds to a polariton-polariton interaction constant of ~ 0.5 μeV μm^{-2} [7].

Supplementary Note 3: Steady-state Bogoliubov excitation spectra

The Bogoliubov perturbation theory is an excellent approximate model to explain the superfluidity and Čerenkov flow even with pulse excitations[8,9]. Here, the polariton interaction energy was much smaller than the polariton splitting between upper and lower polariton branches, so the mixing between LP and UP was very weak. Also, we only researched the LP with resonant excitation, so a simplified model only considering the lower polariton was chosen to solve the Bogoliubov excitation spectrum[7,8]. Considering the resonant excitation case, the wavefunction of the LP branch has a plane-wave form as:

$$\psi(\mathbf{x},t) = \psi_{SS} e^{i(\mathbf{k}_\mathrm{p}\cdot\mathbf{x}-\omega_p t)}$$

The excitation spectrum can be solved from the following equation:

$$\mathcal{L}_k \mathcal{U}_k = \hbar\omega_k \mathcal{U}_k ,$$

where $\mathcal{U}_k$ is the perturbation vector given as

$$\mathcal{U}_k = \left[\delta\psi(\mathbf{x},t), \delta\psi^*(\mathbf{x},t)\right]^T .$$

$\mathcal{L}_k$ is the Bogoliubov operator defined as:

$$\mathcal{L}_k = \begin{pmatrix} \omega_{LP}(k) + 2gn_p - i\gamma_{LP}/2 & gn_p \\ -gn_p & 2\omega_p - \omega_{LP}(2k_p - k) - 2gn_p - i\gamma_{LP}/2 \end{pmatrix},$$

where g is the polariton-polariton interaction constant and n_p is the polariton density. The Bogoliubov modes can be obtained by solving the eigenvalues. For each *k*, the spectrum has two branches $\omega_{LP}^{\pm}$, which are the image of each other under the simultaneous transformations $k \rightarrow 2k_p - k$. For simplicity, only the positive LP Bogoliubov mode is shown in Fig. 5.

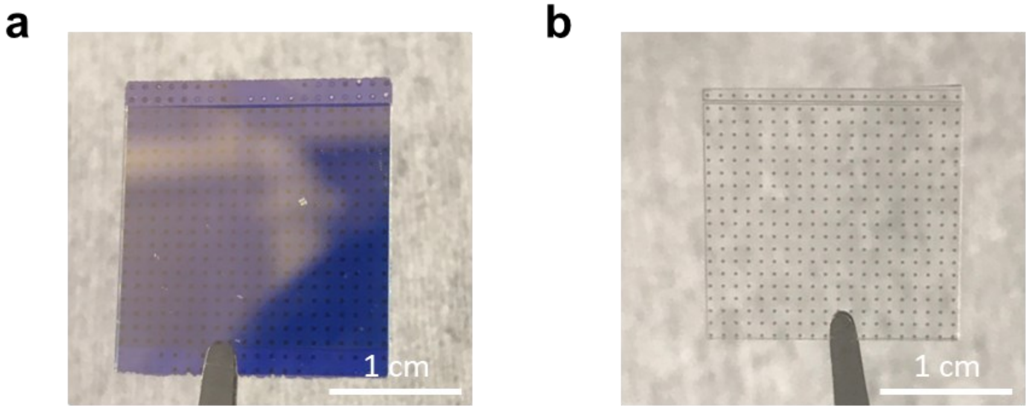

Supplementary Fig. 1 | Images of fabricated chips (2 cm × 2 cm) for crystal growth. **a,** Bonded DBR chips. **b,** Bonded transparent quartz chip without DBR mirrors illustrated for better visualization of the gold pillar arrays.

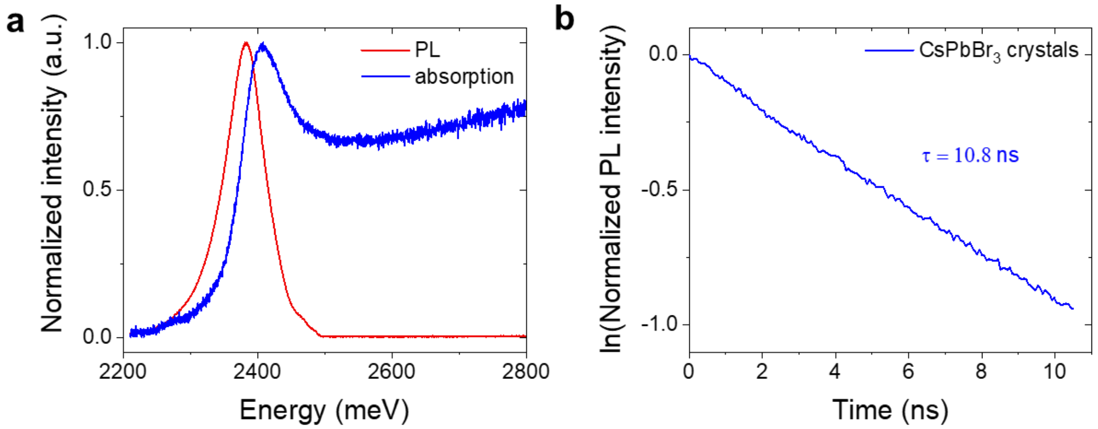

Supplementary Fig. 2 | Optical characterization of $CsPbBr_3$ perovskite crystals at room temperature. Here, the $CsPbBr_3$ single crystals were synthesized in a quartz chip without DBR mirrors. **a,** The absorption and PL emission of a typical $CsPbBr_3$ single crystal shows a strong and stable excitonic peak at ~2406 meV. **b,** The lifetime is measured as 10.8 ns, which implies superior excitonic quality.

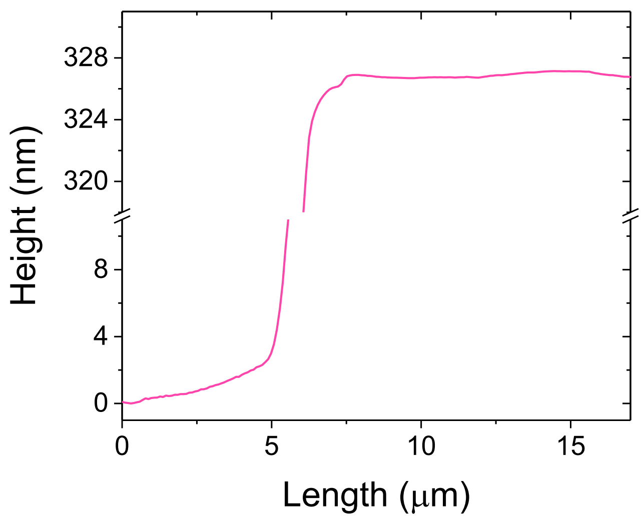

Supplementary Fig. 3 | The thickness profile of $CsPbBr_3$ crystals characterized by atomic force microscopy (AFM). The after-grown microcavity was opened. The crystal thickness was measured by AFM. The thickness is ~326.9±0.3 nm over more than 10 μm, revealing exceptional thickness uniformity.

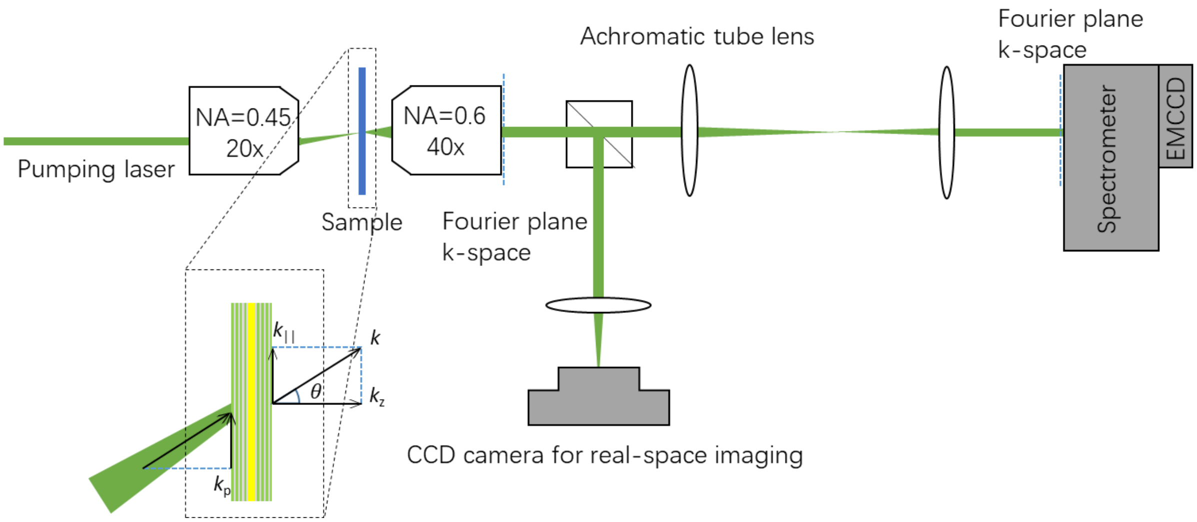

Supplementary Fig. 4 | Experimental setup for room-temperature polariton fluid experiments. The injection momentum k_p is tuned by shifting the laser beam. The in-plane wavevector $k_{\|}$ is determined by $k_{\|} = (\omega / c)\sin\theta$, where ω is the angular frequency, c is the speed of light in vacuum. Thus, the images of polariton emission in transmission configuration in real and momentum space can be observed simultaneously. A Fourier imaging configuration was used to obtain k-space imaging.

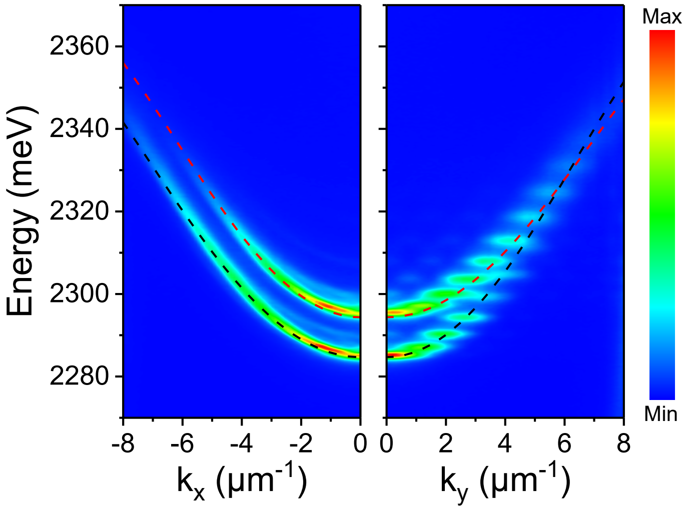

Supplementary Fig. 5 | Angle-resolved PL of another $CsPbBr_3$ microwire with a width of ~4.3 μm. The dashed lines are the fittings of the two branches. The two branches cross at the diabolic points at k_y = 5.9 μm^{-1}. Parameters were fitted as E_0 = 2333.25±10.17 meV, m = 0.13± 0.01 meV/(μm/(ps))^2, α = 6.6±0.55 meV, β = 0.18±0.02 meV μm^2, Ω = 139.91±16.67 meV.

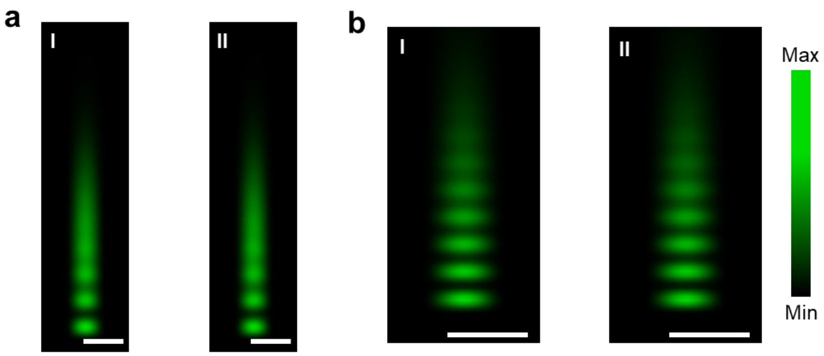

Supplementary Fig. 6 | Numerically simulated real-space images without polariton-polariton interaction. All the other parameters are the same as Fig. 2-3, except constant g = 0 for superfluidity (**a**) and supersonic cases (**b**). Scale bars, 2 μm. To verify that the superfluidity and supersonic behaviors are from the nonlinear polariton-polariton interaction, the interaction constant g is set to zero with other parameters unchanged in the simulation. The interference patterns with high pumping power in II are identical to the low power cases in I for superfluidity and supersonic cases, only with a higher polariton density.

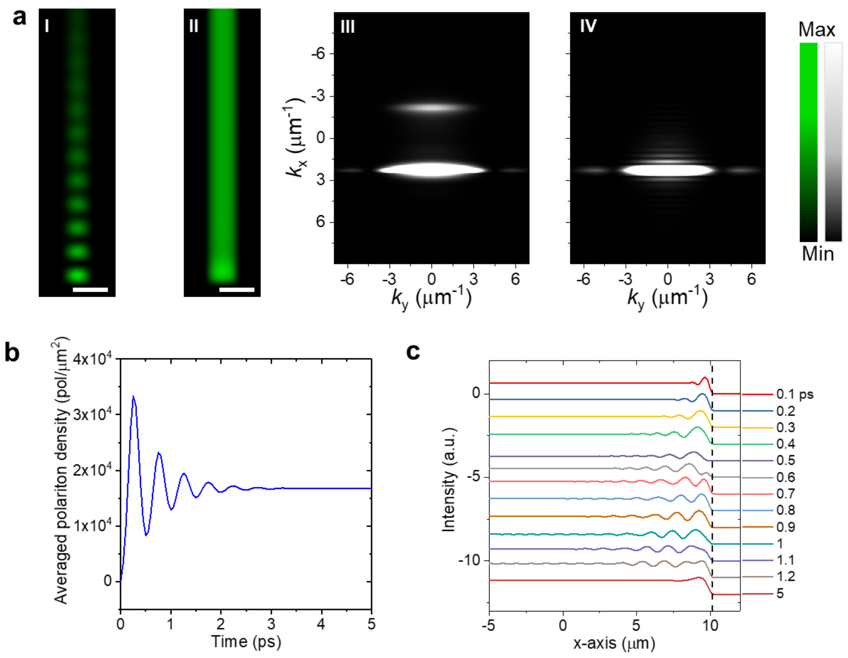

Supplementary Fig. 7 | Numerically simulated steady-state superfluid in perovskite microwire under continuous wave (CW) excitation. The simulation parameters are the same with Fig. 2b except for a large flat top CW pumping laser spot that uniformly covers the whole simulation regime (while in the main text, it is a finite-size gaussian beam). **a**-I-II, Simulated steady-state images in real-space before and after superfluidity transition, respectively. **a**-III-IV, The simulated saturated images in momentum space corresponding to a-I and a-II, respectively. The homogeneous polariton density in real space and the significantly reduced back-reflection signal at ~ -2.3 μm^{-1} illustrate the superfluidity state. The residue scattering in a-IV comes from the finite broadening of the polariton mode induced non-resonant excitation[7]. **b**, Time-dependent averaged polariton density. The pumping laser intensity is set to constant from t = 0 ps. The polariton density oscillates at first and gradually reaches a steady state. **c**, Time-dependent normalized polariton density profiles along the x-axis. The dashed line shows the physical flat end location of the microwire in the simulation. The polariton density backgrounds are normalized and shifted for clarity. The small fluctuation of polariton density at the edge of the microwire shows the reflection of superfluid in the healing length domain. Scale bars, 2 μm.

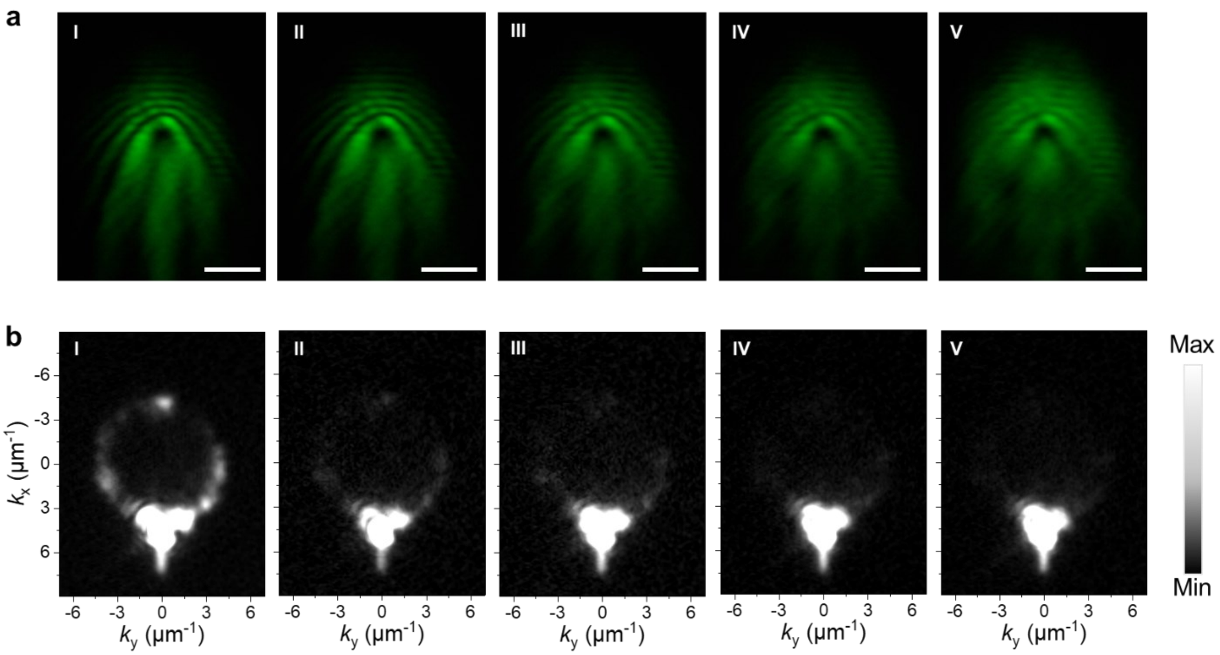

Supplementary Fig. 8 | Experimental real- and k-space images of Čerenkov behavior. a-I-V, More images of the Čerenkov behavior corresponding to Fig. 4a with pumping power in the cavity of 0.07, 1.33, 2, 3.34, and 6 μJ cm^{-2}, respectively. **b**-I-V, The experimental saturated images of the momentum distributions corresponding to **a**-I-V.

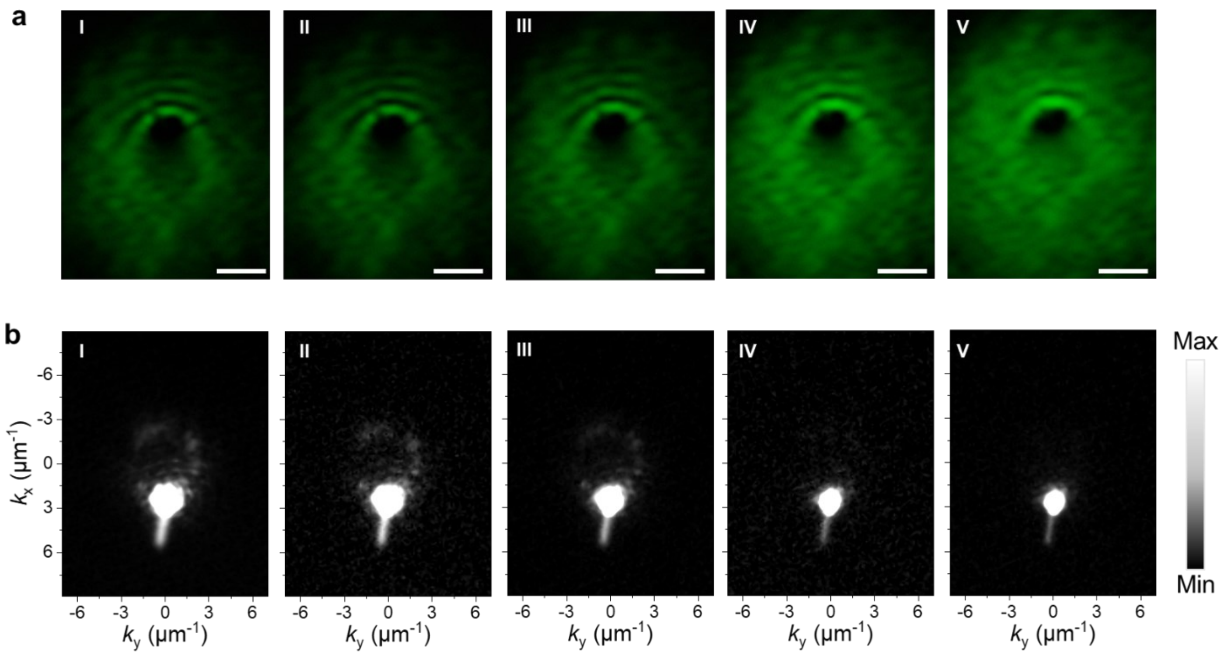

Supplementary Fig. 9 | Experimental real- and k-space images of superfluidity behavior. a-I-V, More images of the superfluidity behavior corresponding to Fig. 4c with pumping power in the cavity of 0.06, 0.56, 1.68, 2.8, and 5.6 μJ cm^{-2}, respectively. **b**-I-V, The experimental saturated images of the momentum distributions corresponding to **a**-I-V.

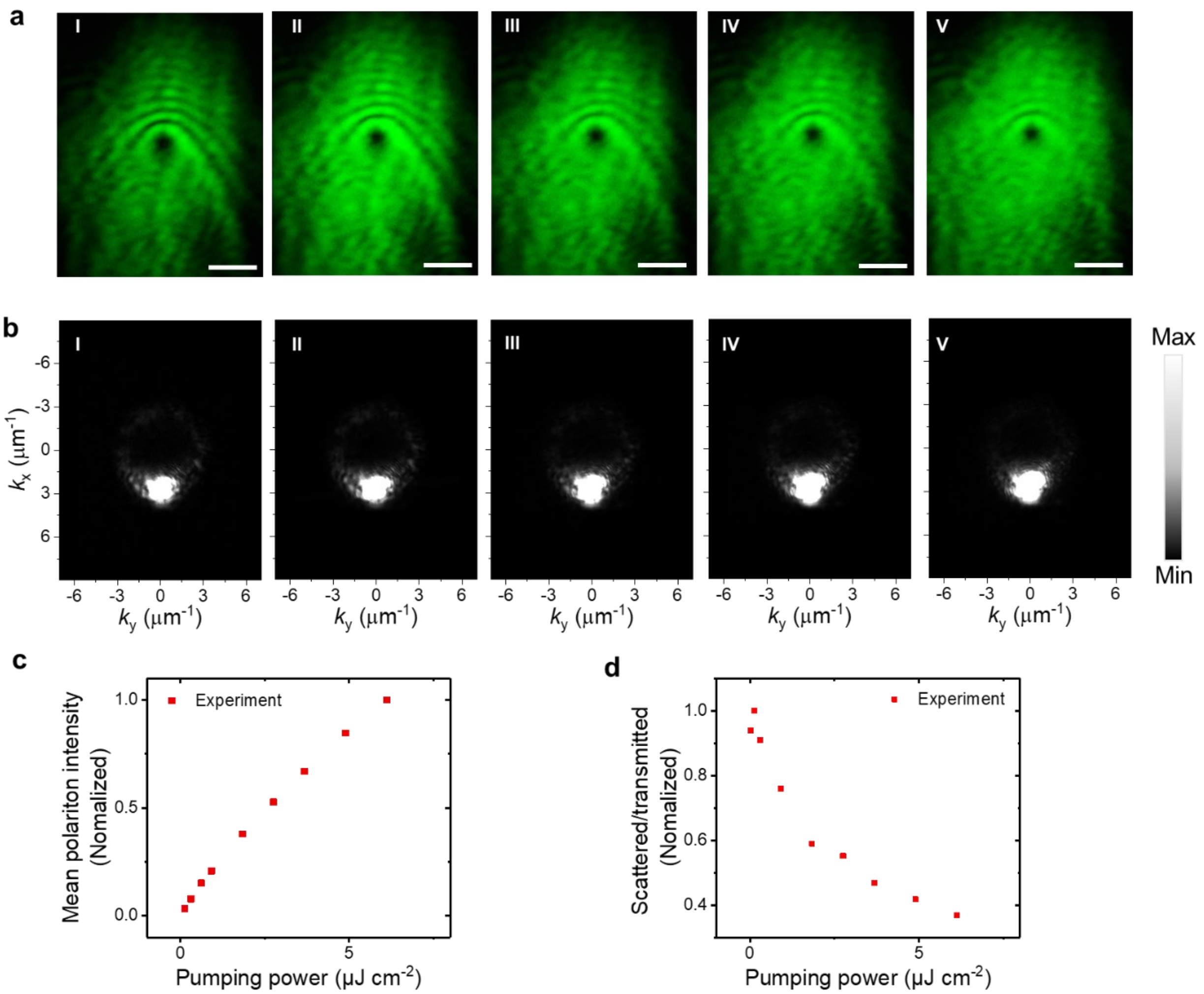

Supplementary Fig. 10 | Experimental superfluidity behavior of a natural defect with injection momentum $k_p = 2.6\ \mu m^{-1}$. Scale bars, 5 μm. **a**-I-V, The real-space images with pumping power in the cavity of 0.12, 0.92, 2.76, 3.67, and 6.12 μJ cm^{-2}, respectively. It appears with more random defects around the natural defect, since the crystal quality tends to be a bit worse in the area near where the natural defect occurs. **b**-I-V are the corresponding momentum-space images. **c**, The experimental normalized mean polariton intensity as a function of pumping power. **d**, The experimental normalized ratio of scattering light to transmitted light as a function of pumping power.

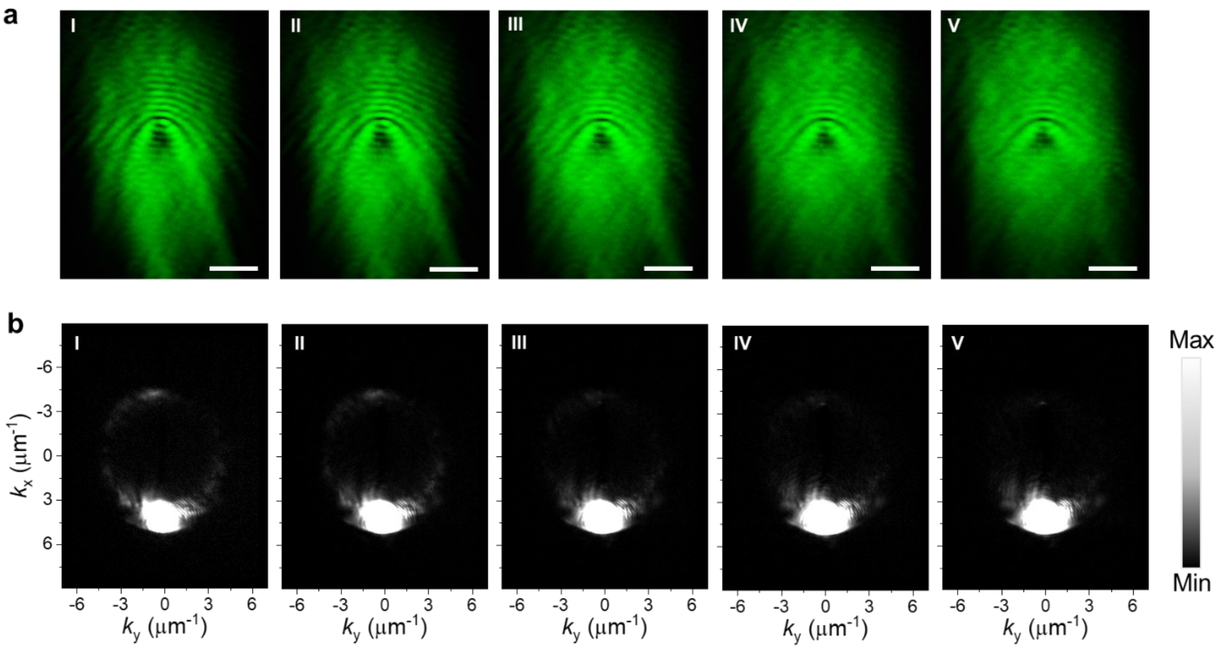

Supplementary Fig. 11 | Experimental Čerenkov behavior of the same natural defect in Fig. S10 with injection momentum k_p = 4.3 μm^{-1}. Scale bars, 5 μm. **a**-I-V, The real-space images with pumping power in the cavity of 0.05, 1.21, 3.21, 5.35 and 8.03 μJ cm^{-2}, respectively. **b**-I-V are the corresponding momentum-space images. The injection laser was partially blocked by the edge of the objective in the pumping part, as shown in the k-space images in Fig. **b**.

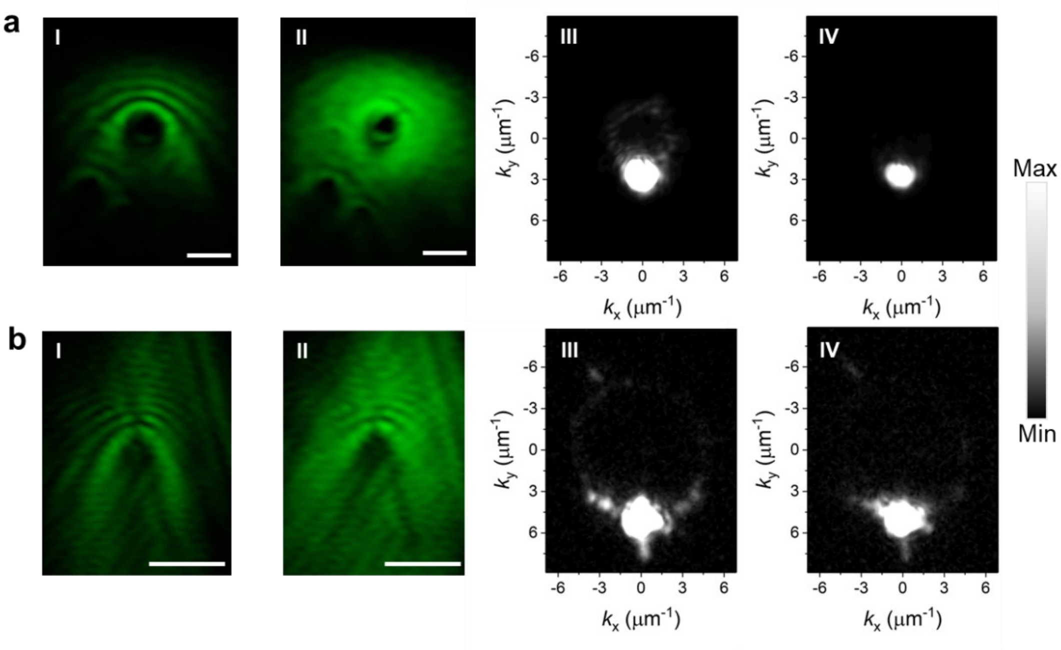

Supplementary Fig. 12 | Experimental superfluidity and Čerenkov behaviors of other natural defects with injection momentum k_p = 2.3 μm^{-1} and k_p = 4.8 μm^{-1} respectively. Scale bars, 5 μm. **a**-I-II, The real-space and images with pumping power in the cavity of 0.08 and 3.3 μJ cm^{-2}, respectively. **a**-III-IV are the corresponding momentum-space images. The superfluidity is demonstrated by vanishing interference fringes and Rayleigh scattering rings at high pumping power (a-II and IV). It appears with two other uncontrolled defects on the bottom left of the natural defect, as the crystal quality tends to be worse in the area where the natural defect occurs. **b**-I-II are the real-space images with pumping power in the cavity of 0.09 and 8.75 μJ cm^{-2}, respectively. **b**-III-IV are the corresponding momentum-space images. Here, the natural defect is ~ 1 μm in diameter. It appears with more random defects around the natural defect, since the crystal quality tends to be a bit worse in the area near where the natural defect occurs.

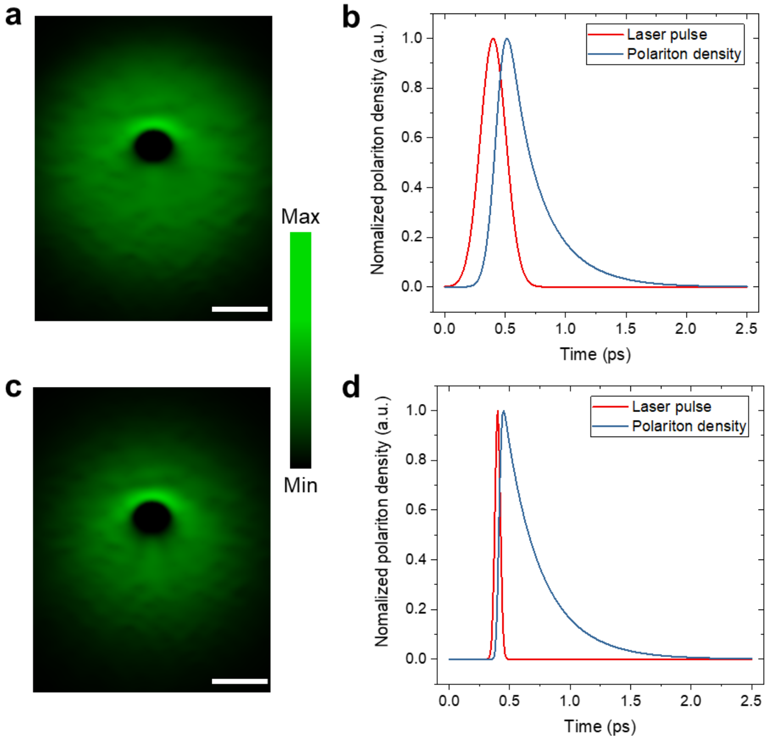

Supplementary Fig. 13 | Simulated superfluidity behaviors with different laser pulse durations. a and **b** are the time-averaged real-space image and time-evolution of the normalized integral polariton density with 250-fs laser pulse excitation. The polariton density decays slower than the laser pulse, which demonstrates a free evolution of the polariton fluid. This effect is even more obvious for a 50-fs laser pulse duration in **c** and **d**. Scale bars, 5 μm. The time-averaged real-space images in these two conditions are quite similar. There is new theory work that the experimental superfluidity under continuous-wave excitation can be understood as a rigid state rather than superfluidity due to the external pump laser phase-locking[10]. However, it is worth pointing out that the phase-locking effect does not exist in pulse excitation cases. This is because the polariton can freely evolve for a more significant proportion of its life after the laser pulse excitation (**b** and **d**). In addition, there is no stationary state under pulse excitation, which is quite different from the continuous wave excitation cases. The generation and flow of vortex pairs can be observed numerically behind the defect, which is a signature of phase evolution and consistent with previous reports[8,9,11].

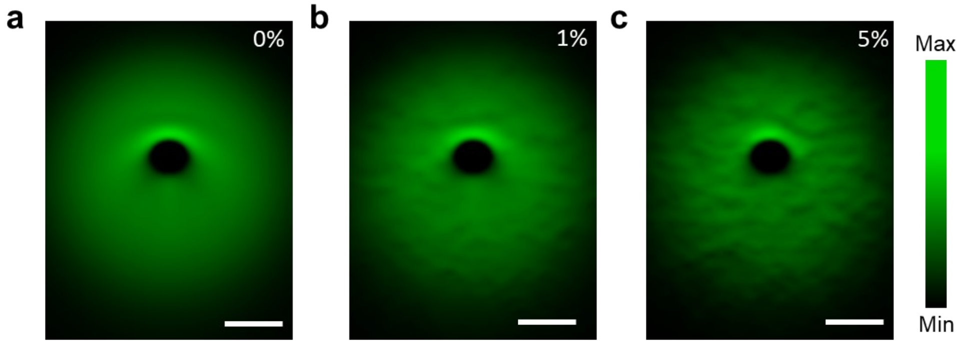

Supplementary Fig. 14 | Simulated superfluidity behaviors with random defect (uniform distribution, not gaussian distribution) spatial pixels ratios. a-c, simulated time-averaged real-space images of superfluidity behaviors with random defect spatial ratios from 0, 1% and 5%, respectively. Scale bars, 5 μm. A uniform random distribution between 0-5 meV exciton energy fluctuation (~ 1.2 nm PL peak position shift) was introduced to account for the small PL inhomogeneous shift in the experiments. A 1% ratio (**b**) fits the experimental data (Fig. 4**d**-II) the best, especially around the defect. These simulation results further justify the exceptional homogeneity of the synthesized perovskite crystals from nanocavities.

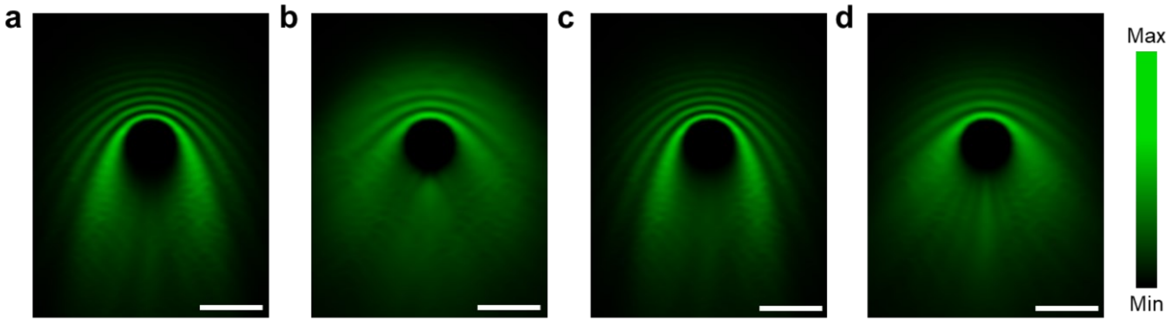

Supplementary Fig. 15 | Simulated Čerenkov behaviors of a large defect with different laser pulse durations. **a-b**, the time-averaged real-space images with low and high pumping power with a 250-fs laser pulse excitation, respectively. **c-d**, the time-averaged real-space images with low and high pumping power with a 35-fs laser pulse excitation, respectively. The increased aperture angles and linear wavefronts at high polariton density in **b** and **d** show the Čerenkov behaviors, regardless of defects sizes and pulse excitation duration lengths in perovskite. Thus, the results indicate that previous missing Čerenkov behaviors mainly come from the inferior nonlinearity and stability in organics materials[8]. Scale bars, 5 μm.

References

1. Dirin, D. N., Cherniukh, I., Yakunin, S., Shynkarenko, Y. & Kovalenko, M. V. Solution-Grown CsPbBr3Perovskite Single Crystals for Photon Detection. *Chem. Mater.* **28**, 8470–8474 (2016).

2. Terças, H., Flayac, H., Solnyshkov, D. D. & Malpuech, G. Non-Abelian gauge fields in photonic cavities and photonic superfluids. *Phys. Rev. Lett.* **112**, 1–5 (2014).

3. Gianfrate, A. *et al.* Measurement of the quantum geometric tensor and of the anomalous Hall drift. *Nature* **578**, 381–385 (2020).

4. Voronych, O., Buraczewski, A., Matuszewski, M. & Stobińska, M. Numerical modeling of exciton–polariton Bose–Einstein condensate in a microcavity. *Comput. Phys. Commun.* **215**, 246–258 (2017).

5. Amo, A. *et al.* Superfluidity of polaritons in semiconductor microcavities. *Nat. Phys.* **5**, 805–810 (2009).

6. Wouters, M. & Carusotto, I. Parametric oscillation threshold of semiconductor microcavities in the strong coupling regime. *Phys. Rev. B* **75**, 075332 (2007).

7. Carusotto, I. & Ciuti, C. Probing microcavity polariton superfluidity through resonant rayleigh scattering. *Phys. Rev. Lett.* **93**, 166401 (2004).

8. Lerario, G. *et al.* Room-temperature superfluidity in a polariton condensate. *Nat. Phys.* **13**, 837–841 (2017).

9. Nardin, G. *et al.* Hydrodynamic nucleation of quantized vortex pairs in a polariton quantum fluid. *Nat. Phys.* **7**, 635–641 (2011).

10. Juggins, R. T., Keeling, J. & Szymańska, M. H. Coherently driven microcavity-polaritons and the question of superfluidity. *Nat. Commun.* **9**, 4062 (2018).

11. Sanvitto, D. *et al.* All-optical control of the quantum flow of a polariton condensate. *Nat. Photonics* **5**, 610–614 (2011).
